# A study on AI-enabled course development, AI proficiency, and first-year students’ academic and psychological adjustment

**DOI:** 10.3389/fpsyg.2026.1909803

**Published:** 2026-09-22

**Authors:** Jun Zhang, Yushi Yin

**Affiliations:** Yangzhou Polytechnic Institute, Yangzhou, Jiangsu, China

**Keywords:** AI proficiency, AMOS, bootstrap mediation, development of digital and AI courses, hierarchical regression, learning adaptation, mental health, process

## Abstract

**Background:**

The rapid integration of digital technologies and generative artificial intelligence (AI) into higher education has reshaped first-year students’ learning experiences and self-reported psychological adjustment. Using a cross-sectional self-report design, this study examined the statistical associations among AI-enabled course development, AI proficiency, academic adjustment, and a composite indicator of self-reported psychological adjustment.

**Methods:**

A cross-sectional self-report survey was conducted among 551 first-year university students from a single institution. Descriptive statistics, Pearson correlations, multiple and hierarchical regressions, Bootstrap mediation analysis (PROCESS), moderated mediation analysis, and structural equation modeling (AMOS) were employed to examine the hypothesized associations. Causal inference is not warranted given the cross-sectional and self-report nature of the data.

**Results:**

Students reported high satisfaction with AI-enabled course development and strong acceptance of AI learning tools, with over 80% recognizing AI’s learning efficiency and actively using AI for study. However, self-reported indicators of psychological strain were endorsed by more than two-thirds of the sample; these items are descriptive self-report indicators and do not constitute clinical diagnoses. Regression analyses showed that course development satisfaction and AI proficiency were statistically and positively associated with emotional management, stress coping, and academic adjustment. Hierarchical regression demonstrated that these variables substantially improved model explanatory power, with the highest explained variance observed for academic adjustment (*R*^2^ = 0.240). Mediation analysis indicated that AI proficiency partially mediated the association between course development satisfaction and self-reported psychological adjustment (indirect effect = 0.124, 95% CI [0.076, 0.181]). Moderation analysis revealed that AI-related usage pressure attenuated the positive association between AI proficiency and self-reported psychological adjustment (moderation index = −0.051, 95% CI [−0.095, −0.017]). Structural equation modeling showed acceptable model fit (*χ*^2^/df = 2.31, CFI = 0.956, TLI = 0.941, RMSEA = 0.049, SRMR = 0.041).

**Conclusion:**

In this cross-sectional sample, AI-enabled course development was statistically and positively associated with first-year students’ self-reported psychological adjustment, both directly and indirectly through self-reported AI proficiency. Excessive self-reported AI-related stress attenuated these positive associations. Because the design is cross-sectional and self-report-based, these findings should be interpreted as statistical associations rather than causal effects. Universities may consider strengthening AI literacy training, optimizing course design, managing technology-related workload, and providing psychological support to maximize the potential benefits of AI-integrated education; future longitudinal and multi-institution studies are needed to test these associations more rigorously.

## 1 Introduction

Artificial intelligence (AI) technology, particularly generative AI tools, is profoundly transforming university curriculum design, knowledge acquisition methods, learning assessment mechanisms, and teacher-student interaction models. For first-year college students, this transformation is not merely a technological update; rather, it is closely intertwined with shifts in learning styles, the establishment of professional identity, the management of academic stress, and self-reported psychological adjustment. First-year students must not only adapt to the characteristics of university courses—which emphasize autonomy, feature open knowledge systems, and employ diverse assessment methods—but also learn to understand and utilize digital platforms, intelligent learning tools, online resources, and AI-assisted systems. Consequently, AI-enabled course development has become a significant contextual factor that may be associated with students’ academic adjustment and self-reported psychological adjustment. Because the evidence summarized below is largely cross-sectional and self-report-based, the present study interprets all observed associations as statistical and does not claim causal effects.

Traditional research on freshman adaptation typically focuses on factors such as learning motivation, peer support, teacher-student relationships, adjustment to campus life, and psychological resilience. With the integration of AI technology into the classroom, however, both the resource structure and the stress structure within the adaptation process appear to have shifted. Course platforms, AI teaching assistants, intelligent feedback, automated retrieval, and generative writing tools can enhance learning efficiency and knowledge accessibility, but they may also be associated with information overload, tool dependency, uncertainty regarding academic standards, anxiety about technical operations, and increased task load. In other words, AI is not inherently synonymous with learning support; it can be more reliably transformed into students’ learning capabilities and self-reported psychological benefits under conditions where course objectives are clear, faculty guidance is sufficient, usage boundaries are well-defined, and evaluation criteria are transparent. The “AI-enabled course development” examined in this study does not refer solely to the use of AI software, but rather to a course ecosystem jointly constituted by course content, teaching resources, digital platforms, AI tools, instructor feedback, normative guidance, and learning assessment. Students’ satisfaction with this ecosystem may shape their perception of the course’s comprehensibility, feasibility, and sense of control. Higher satisfaction with course development has been associated with more stable learning expectations and a stronger sense of learning control, which in prior cross-sectional work has been related to lower adaptation anxiety and better self-reported psychological adjustment. At the same time, the level of AI proficiency has been proposed as a key mechanism linking course development to self-reported psychological adjustment. Even when universities provide abundant AI resources and digital platforms, if students cannot understand the functional boundaries of AI tools, effectively screen and verify AI outputs, or apply AI to learning planning and problem-solving, the supportive association of course development with self-reported psychological adjustment may be difficult to fully realize. Conversely, when students report higher AI proficiency, course resources may be more readily transformed into practical learning strategies, making it easier for students to gain a sense of competence, self-efficacy, and academic adjustment. However, AI-related usage pressure may act as a limiting factor in this positive pathway. Some students, although able to use AI tools, report additional stress regarding AI platform tasks, technical specifications, assignment submissions, AI detection, and instructor evaluations. This pressure may attenuate the positive association between AI proficiency and self-reported psychological adjustment, preventing technological competence from fully translating into self-reported psychological benefits. Therefore, when examining the mediating role of AI proficiency, it is essential to simultaneously consider the moderating association of self-reported AI-related stress.

Against this backdrop, this study examines the statistical associations among first-year students’ self-reported learning adaptation and self-reported psychological adjustment in the context of AI-enabled course development from the perspective of “environmental support—competence transformation—self-reported psychological outcomes.” Unlike previous studies that examined AI adoption or learning anxiety separately, this study integrates course-level support, the self-reported internalization of AI competencies, and stress-related boundary conditions into a single explanatory framework. The study further employs hierarchical regression, Bootstrap mediation, the PROCESS model, and AMOS structural equation modeling to examine the mechanistic associations among course development satisfaction, self-reported AI proficiency, self-reported AI usage stress, and self-reported psychological adjustment. Given the cross-sectional and self-report design, all interpretations in this manuscript are framed in terms of statistical association rather than causal effect.

## 2 Theoretical framework

### 2.1 Self-determination theory and control-value theory

This study constructs its analytical framework primarily on Self-Determination Theory (SDT) and Control-Value Theory (CVT) (Ryan and Deci, 2000; Pekrun, 2006). SDT proposes that autonomy, competence, and relatedness are fundamental psychological needs and that supportive contexts are associated with higher-quality motivation and wellbeing (Ryan and Deci, 2000). CVT posits that achievement emotions are shaped primarily by learners’ appraisals of control and value (Pekrun, 2006). In AI-supported learning environments, clear course structures and effective guidance may be associated with higher perceived control, while AI proficiency may be associated with stronger perceived competence in completing learning tasks. In generative AI course settings, the structured support provided by course design can be viewed as a key external contextual resource that is statistically associated with learners’ self-reported psychological adjustment by affecting their perceived competence and sense of control. Therefore, it is theoretically plausible that “course support → competence transformation → motivation activation → self-reported psychological outcomes” constitutes a coherent explanatory pathway, the empirical associations of which are tested in the present cross-sectional study.

### 2.2 Relationships among core variables and existing research

#### 2.2.1 Course support and psychological outcomes

Existing research generally reports that a supportive instructional environment is associated with learners’ positive emotional experiences and learning satisfaction. Particularly in higher education settings, structured curriculum design may be associated with reduced learning uncertainty by clarifying learning objectives, providing operational demonstrations, and offering normative guidance, thereby improving learning adaptation. In AI education research, existing evidence indicates that systematic AI curricula are associated with stronger AI comprehension and a greater sense of learning empowerment (Hu and Bentler, 1999; Li et al., 2025; Dang Thu and Nguyen Vu, 2025; Soylu et al., 2025). However, current research largely focuses on cognitive-level outcomes, with insufficient attention paid to psychological safety and emotional experiences.

#### 2.2.2 AI competence as a mediating mechanism

AI literacy or AI proficiency has been proposed as a key mediating variable linking external curriculum support to learning outcomes. Relevant studies indicate that AI competence encompasses not only tool operation skills but also a comprehensive set of abilities, including information evaluation, task decomposition, and awareness of norms (Yu et al., 2026; Zhang et al., 2025; Markus et al., 2025). Existing empirical research has found that AI usage behavior is statistically associated with academic self-efficacy through anxiety levels, and that self-efficacy plays a key mediating role in learning psychological outcomes (Xu et al., 2025). Furthermore, generative AI support has been shown to be indirectly associated with learning anxiety and stress experiences through learners’ self-efficacy (Chen et al., 2024; Chai and Sha, 2025; Schiavo et al., 2024; Mohamud et al., 2026; Alqurni, 2026). Collectively, these studies suggest that AI competence may serve as an important mediator in the “environmental support-psychological outcomes” pathway; however, there remains room for further refinement in its structured measurement and the integration of underlying mechanisms.

#### 2.2.3 The role of motivational mechanisms

Within the SDT framework, an externally supportive environment is associated with the development of autonomous motivation by fulfilling basic psychological needs. Existing research indicates that need-supportive leadership is associated with higher motivation levels and work engagement. In educational contexts, motivation not only shapes behavioral persistence but is also directly associated with the quality of psychological experiences. Meta-analysis results indicate that intrinsic motivation is consistently and positively correlated with positive emotions and satisfaction. Therefore, improvements in self-reported AI proficiency may be associated with stronger perceived control and competence, thereby supporting more adaptive emotional experiences (Pekrun, 2006).

#### 2.2.4 AI-related stress and psychological boundaries

With the widespread adoption of AI technology, technostress and AI-related anxiety have gradually emerged as significant factors associated with student adjustment. Research has found that technical barriers may be associated with reduced technological self-efficacy and increased anxiety (Chen et al., 2024; Schiavo et al., 2024; Cengiz and Peker 2025; Zhang et al., 2025). In higher education settings, academic workload and assessment pressure may further amplify these anxiety responses and be negatively associated with learning motivation and self-reported psychological adjustment (Chen et al., 2024; Schiavo et al., 2024; Cengiz and Peker, 2025; Zhang et al., 2025). Consequently, self-reported AI usage pressure may play a significant moderating role in the statistical association between AI competence and self-reported psychological adjustment.

A comprehensive review of the existing literature reveals several areas warranting further exploration. First, regarding course support, existing research largely remains at the level of general descriptions and has not yet established a unified analytical framework that breaks down AI course support into structural elements such as goal setting, instructional demonstrations, and normative guidance; the operationalization of variables remains insufficient. Second, concerning the mechanism of action of AI competence, most studies still treat it as a static indicator, with few systematically examining it through the continuous process of “competence internalization-motivation formation-self-reported psychological outcomes,” leaving its underlying transformative logic unclear. Third, although AI usage pressure has received attention, its moderating role in the statistical association between AI competence and self-reported psychological outcomes within educational contexts still lacks sufficient empirical support. Finally, existing research samples are predominantly concentrated on general college student populations, and specialized models and localized evidence targeting first-year students-a transitional group-remain relatively scarce. In summary, this study integrates Self-Determination Theory (SDT) and Control-Value Theory (CVT) to construct a systematic analytical framework encompassing “curricular support-competence transformation-motivational mechanisms-self-reported psychological outcomes,” further incorporating self-reported AI usage pressure as a key boundary condition. This framework not only addresses the gap in explaining educational psychological mechanisms amid the rapid development of generative AI but also provides a structured theoretical foundation and a system of testable hypotheses for research on freshman adaptation.

## 3 Research methods

### 3.1 Research design and data sources

This study employed a cross-sectional self-report questionnaire design, targeting first-year students in the School of Architectural Engineering at Yangzhou Polytechnic Institute in Jiangsu Province, China. The survey focused on AI-enabled course development, the use of AI learning tools, learning adaptation, and a composite indicator of self-reported psychological adjustment. A total of 551 valid responses were collected. All data underwent rigorous quality control and preprocessing prior to analysis.

The sample composition indicates that the surveyed students were predominantly male, majoring in engineering, and living on campus. Males accounted for 81.85% (*n* = 451) and females 18.15% (*n* = 100); engineering students accounted for 76.41% (*n* = 421) and students in other majors 20.51% (*n* = 113); 90.56% (*n* = 499) lived on campus; and 28.68% (*n* = 158) served as class officers or members of student organizations. This sample reflects the demographic profile of the School of Architectural Engineering at the host institution; the implications of this single-institution, gender-skewed, and discipline-specific sample are discussed in the Limitations section (Figure 1).

**Figure 1:**
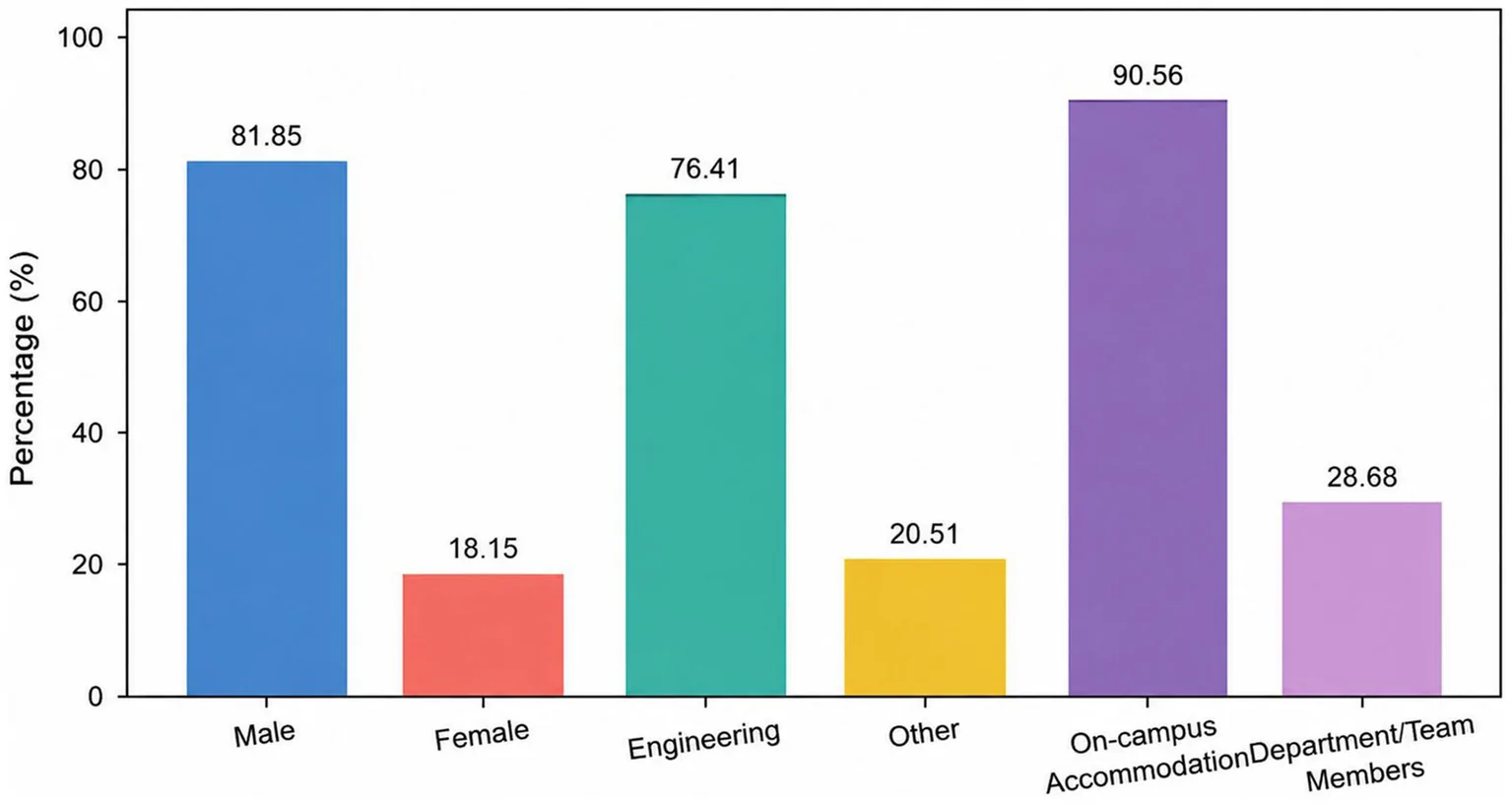
Key proportions of the sample structure.

### 3.2 Measurement model and reliability and validity tests

To ensure the construct validity of the measurement instrument, this study adopted a two-stage analytical strategy: first, confirmatory factor analysis (CFA) was conducted, followed by the development of a structural equation model (Zhang et al., 2025; Chen et al., 2024; Cengiz and Peker 2025).

1. Confirmatory Factor Analysis (CFA)

AMOS 24.0 was used to test a three-factor measurement model comprising course development satisfaction, AI proficiency, and self-reported psychological adjustment. Model fit indices were evaluated using the following criteria: *χ*^2^/df < 3.0, CFI > 0.90, TLI > 0.90, RMSEA < 0.08, and SRMR < 0.08. The three-factor model fit the data well (*χ*^2^/df = 2.31, CFI = 0.956, TLI = 0.941, RMSEA = 0.049, SRMR = 0.041), supporting the structural distinctiveness of the latent constructs.

1. Reliability

This study used Cronbach’s *α* to assess the internal consistency of the scale. The *α* values for all core variables were greater than 0.70, indicating that the scale possesses a good level of reliability. Composite reliability (CR) was also calculated, with results consistently exceeding 0.70, further validating the stability of the scale.

Table 1 reports the detailed psychometric properties of the four core constructs, including the number of items per construct, factor loadings, Cronbach’s *α*, composite reliability (CR), average variance extracted (AVE), and the square root of AVE alongside the inter-construct correlations used to assess discriminant validity. All Cronbach’s *α* values were greater than 0.80, all CR values exceeded 0.85, all AVE values exceeded 0.50, and the square root of AVE for each construct was larger than its correlations with the other constructs, indicating adequate reliability, convergent validity, and discriminant validity.

1. Construct validity and discriminant validity

**Table 1:** Reliability, convergent validity, and discriminant validity of the measurement model.

Construct	Items (*n*)	Factor loading range (mean)	Cronbach’s *α*	CR	AVE	(AVE)^1/2^
Course development satisfaction	6	0.71–0.85 (0.78)	0.91	0.92	0.62	0.79
Self-reported AI proficiency	6	0.65–0.83 (0.74)	0.88	0.89	0.58	0.76
Self-reported psychological adjustment	9	0.62–0.81 (0.72)	0.90	0.91	0.55	0.74
Self-reported AI usage pressure	4	0.74–0.86 (0.80)	0.89	0.90	0.60	0.77

*α*, Cronbach’s alpha; CR, composite reliability; AVE, average variance extracted; (AVE)^1/2^, square root of AVE (diagonal values in the Fornell–Larcker matrix). Discriminant validity: (AVE)^1/2^ for each construct exceeded its largest correlation with any other construct (range of inter-construct *r* = −0.32–0.46); all Heterotrait-Monotrait (HTMT) ratios < 0.85, supporting discriminant validity.

Convergent validity was assessed using the Average Variance Extracted (AVE) and standardized factor loadings. The AVE values for all constructs were greater than 0.50 (course development satisfaction: 0.62; AI proficiency: 0.58; self-reported psychological adjustment: 0.55; AI usage pressure: 0.60), and all standardized factor loadings exceeded 0.60, indicating that the indicators effectively explained the variance of their latent constructs. Discriminant validity was assessed using the Fornell-Larcker criterion. The square root of AVE for each construct was greater than its correlation with any other construct, and the Heterotrait-Monotrait (HTMT) ratio was below 0.85 for all construct pairs, supporting discriminant validity.

### 3.3 Variable measurement

Course development satisfaction was measured with 6 self-developed items adapted from established curriculum-evaluation inventories and informed by the SDT notion of need-supportive teaching. Items captured clarity of course structure, online resource support, instructor AI demonstrations, guidance on appropriate AI use, integration of digital and AI resources, and overall course support. AI proficiency was measured with 6 self-developed items adapted from recent AI literacy scales (e.g., the AICOS framework and the Student AI Literacy Scale), capturing efficiency in using AI tools, AI-assisted learning, task decomposition, information screening and verification, and understanding of AI guidelines. Self-reported psychological adjustment was measured with 9 items adapted from academic-adjustment and emotional-regulation inventories, capturing learning adaptation, emotional management, and stress coping; several negatively worded items (e.g., “I often feel anxious or tense when dealing with study-related demands”) were reverse-coded such that higher composite scores indicate better self-reported adjustment. AI usage pressure was measured with 4 self-developed items capturing the additional burden, uncertainty, and concern associated with AI-platform tasks and AI-related academic requirements. All items used a 5-point Likert response format (1 = Strongly Disagree, 5 = Strongly Agree).

The questionnaire assessed four conceptually distinct self-report constructs: course development satisfaction (a contextual/environmental indicator), AI proficiency (a competence indicator), self-reported psychological adjustment (a composite of academic adjustment, emotional management, and stress coping, treated as a single adjustment composite), and AI usage pressure (a stressor indicator). All items were originally drafted in Chinese and reviewed by three faculty members in educational psychology and instructional design for face validity; minor wording revisions were made following a pilot test with 50 first-year students who were not included in the final sample. Reverse-coded items were recoded before computing composite scores. Because the outcome construct was operationalized through self-report items that tap academic adjustment, emotional management, and stress coping, it is referred to throughout as “self-reported psychological adjustment” rather than “mental health” to avoid implying clinical diagnostic validity (see Table 2).

**Table 2:** Mapping of questionnaire items to theoretical constructs.

Construct	Operational content	Representative item examples	Response format
Course development satisfaction	Course structure clarity; online resource support; instructor AI demonstrations; guidance on appropriate/standardized AI use; integration and overall course support	The course schedule and learning requirements are clear; The instructor provides clear guidance on appropriate AI use.	5-point Likert (1 = Strongly Disagree, 5 = Strongly Agree)
AI proficiency	Efficiency in using AI tools; AI-assisted learning; task decomposition; information screening/verification; understanding of AI-use guidelines	I can use AI tools efficiently to support my learning; I can check and verify AI-generated information before using it.	5-point Likert
Self-reported psychological adjustment	Academic adjustment; emotional management; stress coping; self-reported psychological strain indicators (reverse-coded)	I can adapt to university course requirements and learning methods; I often feel anxious or tense when dealing with study-related demands.	5-point Likert; negatively worded items are reverse-coded (higher score = better adjustment)
AI usage pressure	Additional burden from AI-platform tasks, uncertainty about AI-related requirements, and concerns associated with AI use	Completing tasks on AI platforms creates additional pressure for me.	5-point Likert

### 3.4 Statistical analysis strategy

Analyses were organized around the six pre-registered hypotheses, and each hypothesis was tested with a designated technique to minimize analytical redundancy. (1) Pearson correlations and standardized multiple regressions were used to describe the bivariate and multivariate associations between course development satisfaction, AI proficiency, and the three indicators of self-reported psychological adjustment (H1, H2). (2) Hierarchical regression was used to test the incremental contribution of course development satisfaction and AI proficiency beyond demographic covariates. (3) Bootstrap mediation (PROCESS Model 4, 5,000 resamples) tested the indirect effect of course development satisfaction on self-reported psychological adjustment through AI proficiency (H3, H4). The bootstrap approach for estimating indirect effects followed the resampling-based mediation procedures proposed by Preacher and Hayes (2008). (4) A simple moderation model (PROCESS Model 1) tested whether AI proficiency moderates the association between course development satisfaction and academic adjustment (H5). (5) A moderated mediation model (PROCESS Model 14) tested whether AI usage pressure moderates the indirect association between course development satisfaction and self-reported psychological adjustment through AI proficiency (H6). (6) AMOS structural equation modeling was used to verify the overall model fit and to complement the regression-based mediation and moderation tests with a latent-variable specification. The six hypotheses were mapped to distinct techniques to ensure that each technique addressed a unique theoretical question, and no single substantive finding is reported using more than two statistical techniques.

### 3.5 Research hypotheses

H1: Course development satisfaction is positively associated with self-reported psychological adjustment (academic adjustment, emotional management, and stress coping). H2: AI proficiency is positively associated with self-reported psychological adjustment. H3: Course development satisfaction is positively associated with AI proficiency. H4: AI proficiency partially mediates the positive association between course development satisfaction and self-reported psychological adjustment. H5: AI proficiency moderates the association between course development satisfaction and academic adjustment; the positive association between course development and academic adjustment is expected to be stronger among students with higher AI proficiency. H6: AI usage pressure moderates the association between AI proficiency and self-reported psychological adjustment; the positive association between AI proficiency and self-reported psychological adjustment is expected to be attenuated when AI usage pressure is high.

## 4 Descriptive statistics

### 4.1 Course development satisfaction evaluation

Descriptive results show that students gave the digital and intelligent AI course development a high overall rating. The rate of positive evaluations regarding the course’s assistance in adapting to freshman-year studies was 77.68, 79.49% for clear course scheduling, 81.67% for support with online learning tools, 82.21% for instructors’ use of digital resources, 82.39% for instructors’ guidance on AI standards, 82.76% for the integration of digital and intelligent content into the course, and approximately 82.40% for overall support. These results indicate that surveyed students generally recognize the supportive role of course development in helping them adapt to their studies.

Across all dimensions, the positive evaluation rates for the integration of digital and intelligent content into courses and for instructors’ guidance on AI standards were particularly high. This suggests that students do not reject the introduction of AI into the classroom; on the contrary, they expect instructors to provide clear demonstrations and standardized guidance. The evaluation rate for the course’s assistance in adapting to freshman-year studies was relatively lower, though it still approached 78%. This indicates that further efforts are needed to help freshmen adapt, particularly by integrating course content, learning methods, and the use of AI tools into a clearer learning pathway (Figure 2).

**Figure 2:**
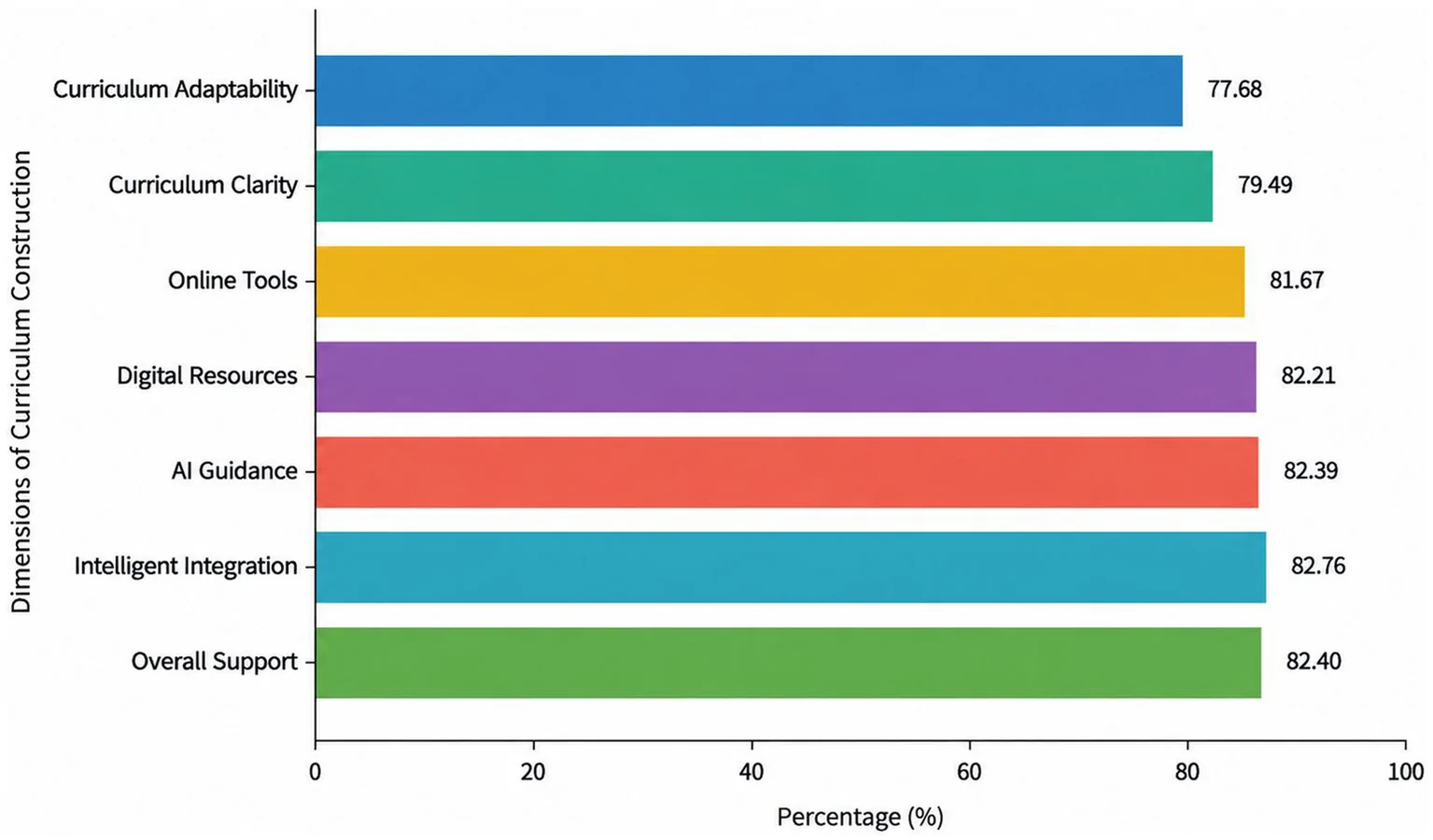
Positive evaluation rate by course development dimension.

### 4.2 Attitudes toward AI learning and proficiency with AI

The results regarding attitudes toward AI learning show that 83.48% of students believe AI can improve learning efficiency, 84.03% indicate they would proactively use digital platforms or AI tools for learning, and 83.31% are more willing to try AI tools after seeing a teacher demonstrate their use. This indicates that AI tools have become part of students’ daily learning practices and have emerged as a significant factor influencing learning methods. However, attitudes toward AI learning are not entirely positive. 73.50% of students worry that overreliance on AI will hinder independent thinking, and 66.97% feel additional pressure when completing tasks on AI platforms. This concern is consistent with recent evidence that personal characteristics and psychological needs may shape AI dependency among university students (Zhong et al., 2024). These findings reveal a dual nature in AI-based learning: on the one hand, AI tools are viewed as resources for efficiency; on the other hand, AI dependency and the pressure of platform tasks also pose new psychological risks (Figure 3).

**Figure 3:**
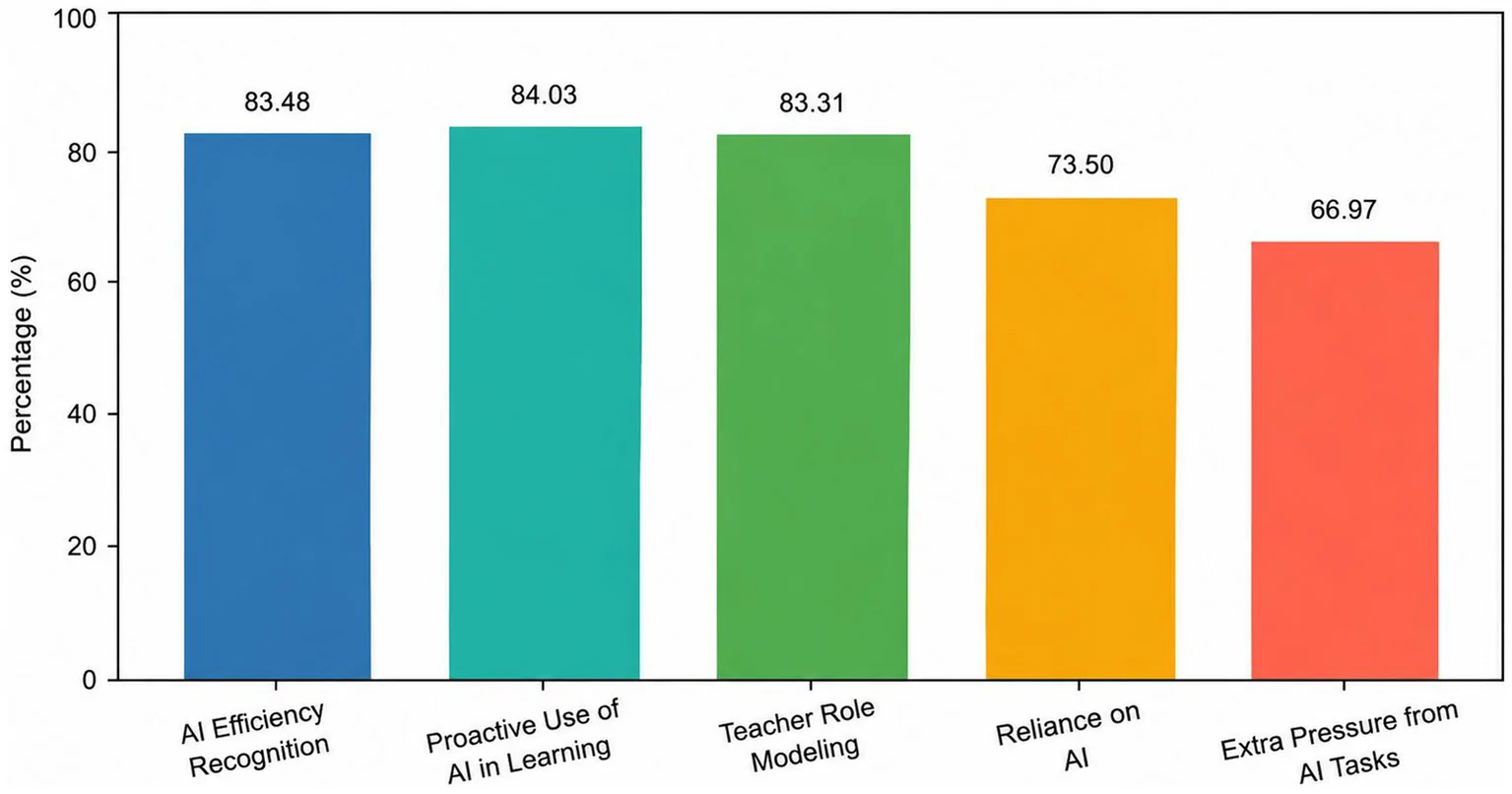
Indicators of attitudes toward AI learning and stress related to AI use.

### 4.3 Academic adjustment and self-reported psychological strain

Self-reported psychological strain indicators show that the surveyed students exhibit a coexistence of positive adjustment and perceived strain. While most students reported being able to adapt to course requirements and AI learning tools to some extent, the proportions endorsing items related to anxiety, irritability, difficulty relaxing, low self-worth, loneliness, and AI-related task pressure were all above 65%. The endorsement rates were 69.51% for anxiety/tension, 68.78% for irritability, 67.88% for difficulty relaxing and stress, 67.70% for low self-worth, 67.52% for loneliness and lack of support, and 66.97% for additional pressure from AI platform tasks. Because these figures were derived from self-report questionnaire items rather than from validated clinical diagnostic instruments, they should be interpreted as self-reported psychological strain indicators or perceived stress-related experiences, not as prevalence estimates of clinically diagnosable mental health disorders.

These results indicate that, in the context of digital and AI course development, first-year students do not simply “accept technology” or “reject technology,” but rather constantly adjust between the benefits of technology and the pressures it imposes. While course development can provide resources, if AI tasks are designed too complexly, evaluation criteria are unclear, or students lack the ability to use the technology properly, the advantages of technology may turn into a psychological burden (Figure 4).

**Figure 4:**
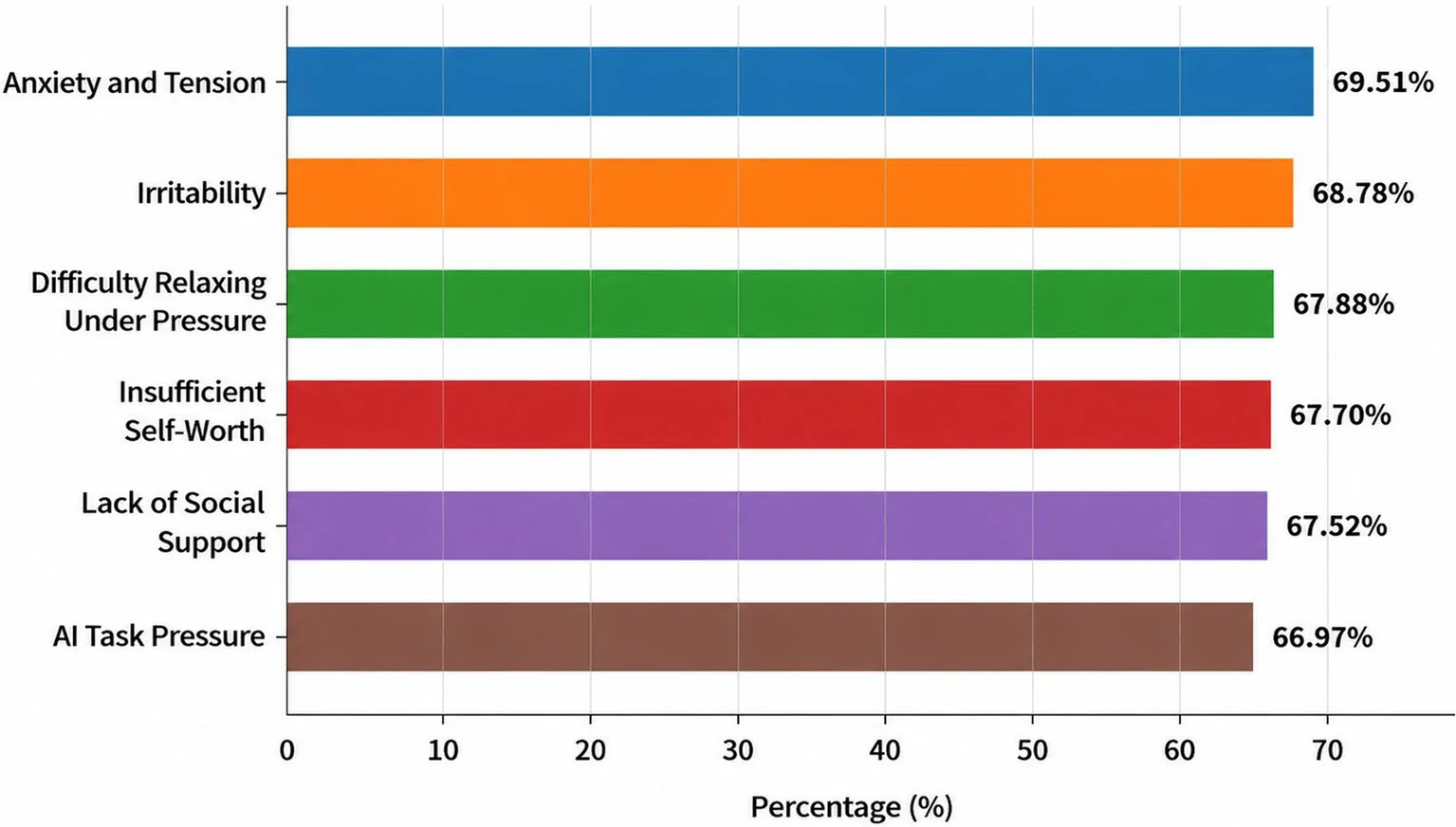
Psychological stress and the incidence of risk experiences.

### 4.4 Summary of descriptive findings

Overall, the descriptive statistics reveal three key findings. First, course development and AI learning tools received high levels of recognition, indicating that AI-enabled course development has a solid foundation of student acceptance. Second, while AI tools are widely viewed as resources for enhancing learning efficiency, concerns about AI dependency and the pressure associated with AI-based assignments are also prominent. Third, self-reported indicators of psychological strain do not automatically decrease with the digitization of courses; rather, they exhibit a coexistence of “high support and high strain.” It is therefore necessary to further examine, through regression, mediation, and moderation models, how course development is associated with students’ self-reported psychological adjustment and what role AI proficiency plays in this association.

## 5 Correlation analysis and multiple regression

### 5.1 Correlation analysis of variables

Correlation analysis was used to preliminarily examine the linear associations among course development satisfaction, AI proficiency, AI usage pressure, and self-reported psychological adjustment. According to the model specifications, course development satisfaction was expected to be positively associated with AI proficiency, emotional management, stress coping, and academic adjustment; AI proficiency was expected to be positively associated with self-reported psychological adjustment; and AI usage pressure was expected to be negatively associated with self-reported psychological adjustment, while potentially exhibiting a complex association with AI proficiency.

Table 3 presents the bivariate correlation matrix.

**Table 3:** Correlation matrix of core variables.

Variables	1	2	3	4	5	6
Course development satisfaction	1.00					
Self-reported AI proficiency	0.46	1.00				
Self-reported AI usage pressure	−0.18	−0.12	1.00			
Self-reported emotional management	0.39	0.35	−0.28	1.00		
Self-reported stress coping	0.35	0.31	−0.32	0.58	1.00	
Self-reported academic adjustment	0.42	0.38	−0.25	0.61	0.57	1.00

### 5.2 Multiple regression analysis

To further examine the independent statistical associations of course development satisfaction and AI proficiency with the three indicators of self-reported psychological adjustment, this study estimated multiple regression models with emotional management, stress coping, and academic adjustment as dependent variables (Figures 5, 6; Table 4).

**Figure 5:**
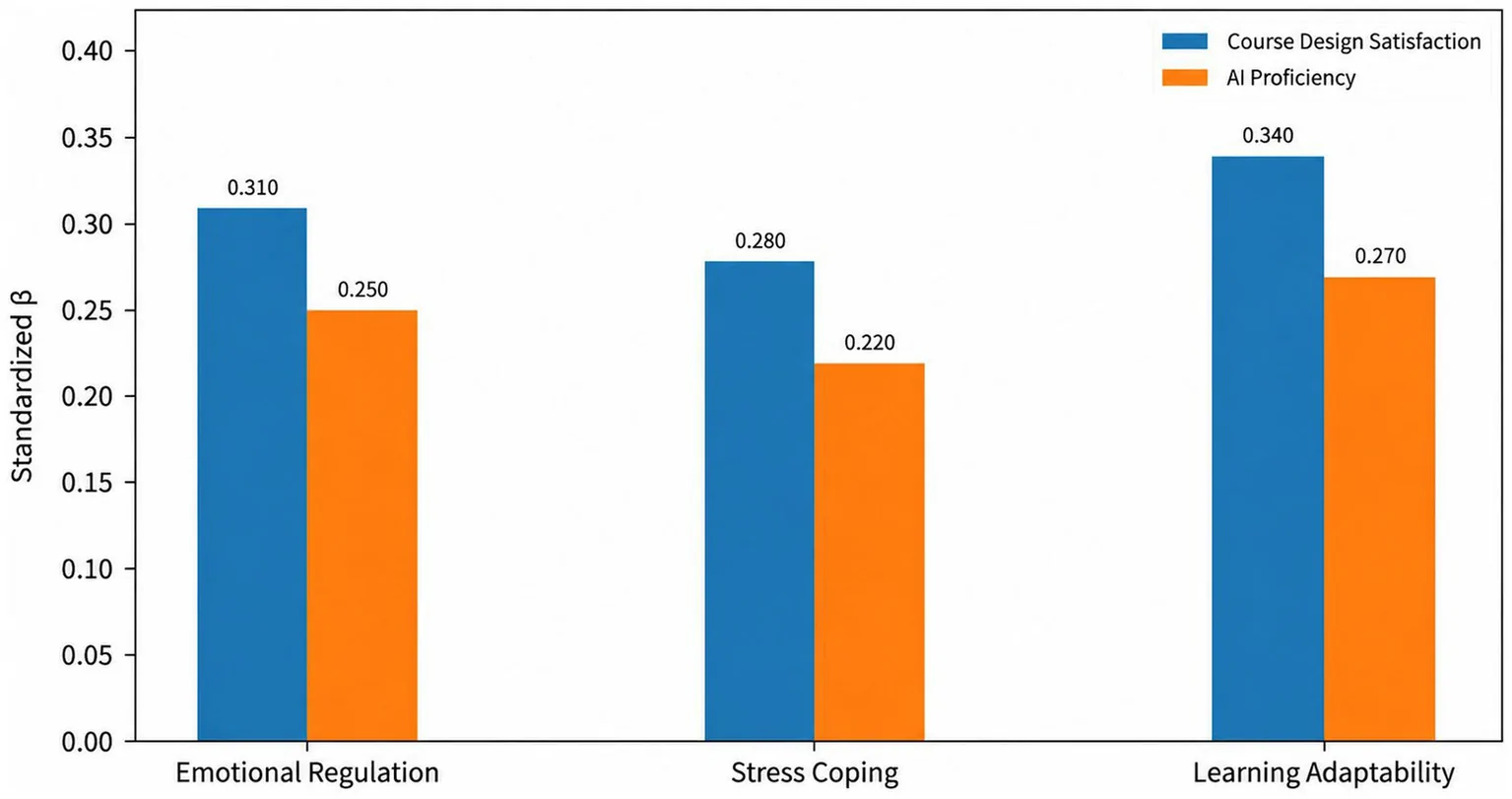
Standardized predictive effects of course development satisfaction and AI proficiency.

**Figure 6:**
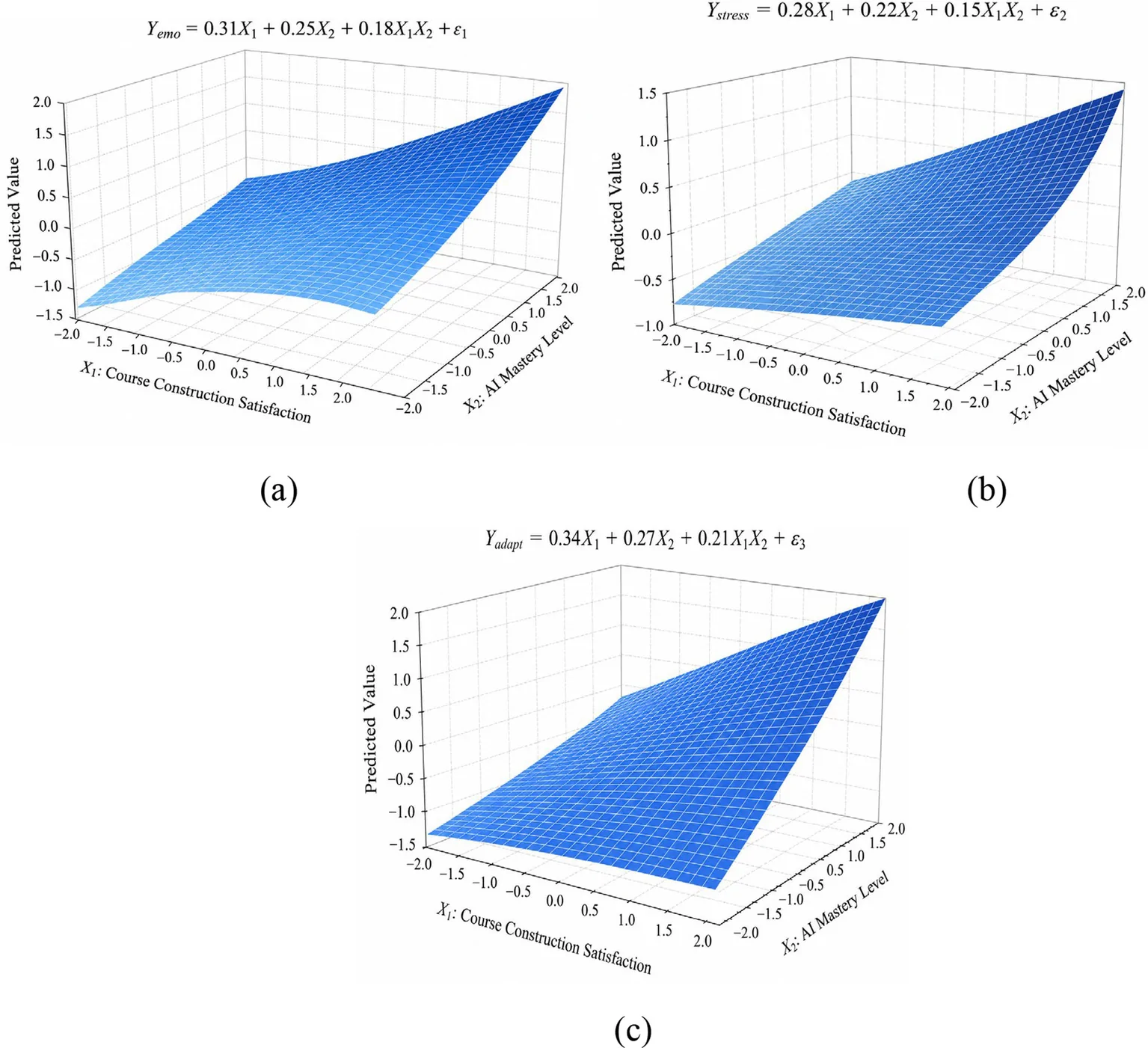
Multiple regression analysis models: **(a)** Emotional regulation model; **(b)** Stress coping model; **(c)** Academic adjustment model.

**Table 4:** Summary of multiple regression results.

Dependent variable	Predictor variables	Standardized *β*	*t*	*p*	*R* ^2^
Self-reported emotional management	Course development satisfaction	0.31	7.85	<0.001	0.21
Self-reported emotional management	Self-reported AI proficiency	0.25	6.32	<0.001	
Self-reported stress coping	Course development satisfaction	0.28	6.45	<0.001	0.18
Self-reported stress coping	Self-reported AI proficiency	0.22	5.71	<0.001	
Self-reported academic adjustment	Course development satisfaction	0.34	8.10	<0.001	0.24
Self-reported academic adjustment	Self-reported AI proficiency	0.27	6.78	<0.001	

*ε*₁, *ε*_2_ and *ε*_3_ is assumed to be independently and identically distributed with zero mean and constant variance, representing all omitted variables and random shocks not explicitly modeled in this study.

### 5.3 Interpretation of basic regression results

These regression results are consistent with H1 and H2. Higher course development satisfaction is associated with better emotional management, stress coping, and academic adjustment; higher AI proficiency is associated with better outcomes on the same three indicators. The learning adjustment model showed the highest *R*^2^, suggesting that course development satisfaction and AI proficiency have the strongest statistical association with learning adaptation in this sample, which is consistent with the developmental characteristics of first-year students who are simultaneously transitioning from high school to university and from traditional to AI-supported learning.

## 6 Advanced statistical analysis and model testing

Building upon the descriptive statistics, correlations, and multiple regressions reported in Section 5, this section presents a series of regression-based and structural-equation-modeling analyses organized around hypotheses H3–H6. The purpose is to examine the indirect effect of course development satisfaction on self-reported psychological adjustment through AI proficiency (H3, H4), and to examine two distinct moderation models: (i) AI proficiency moderating the association between course development satisfaction and academic adjustment (H5), and (ii) AI usage pressure moderating the association between AI proficiency and self-reported psychological adjustment within the mediation model (H6).

### 6.1 Hierarchical regression analysis

Hierarchical regression was used to examine the incremental statistical association of course development satisfaction and AI proficiency with self-reported psychological adjustment after controlling for demographic variables. Model 1 included gender, major category, and housing status as demographic covariates. Model 2 added course development satisfaction to Model 1. Model 3 further added AI proficiency (Figure 7).

**Figure 7:**
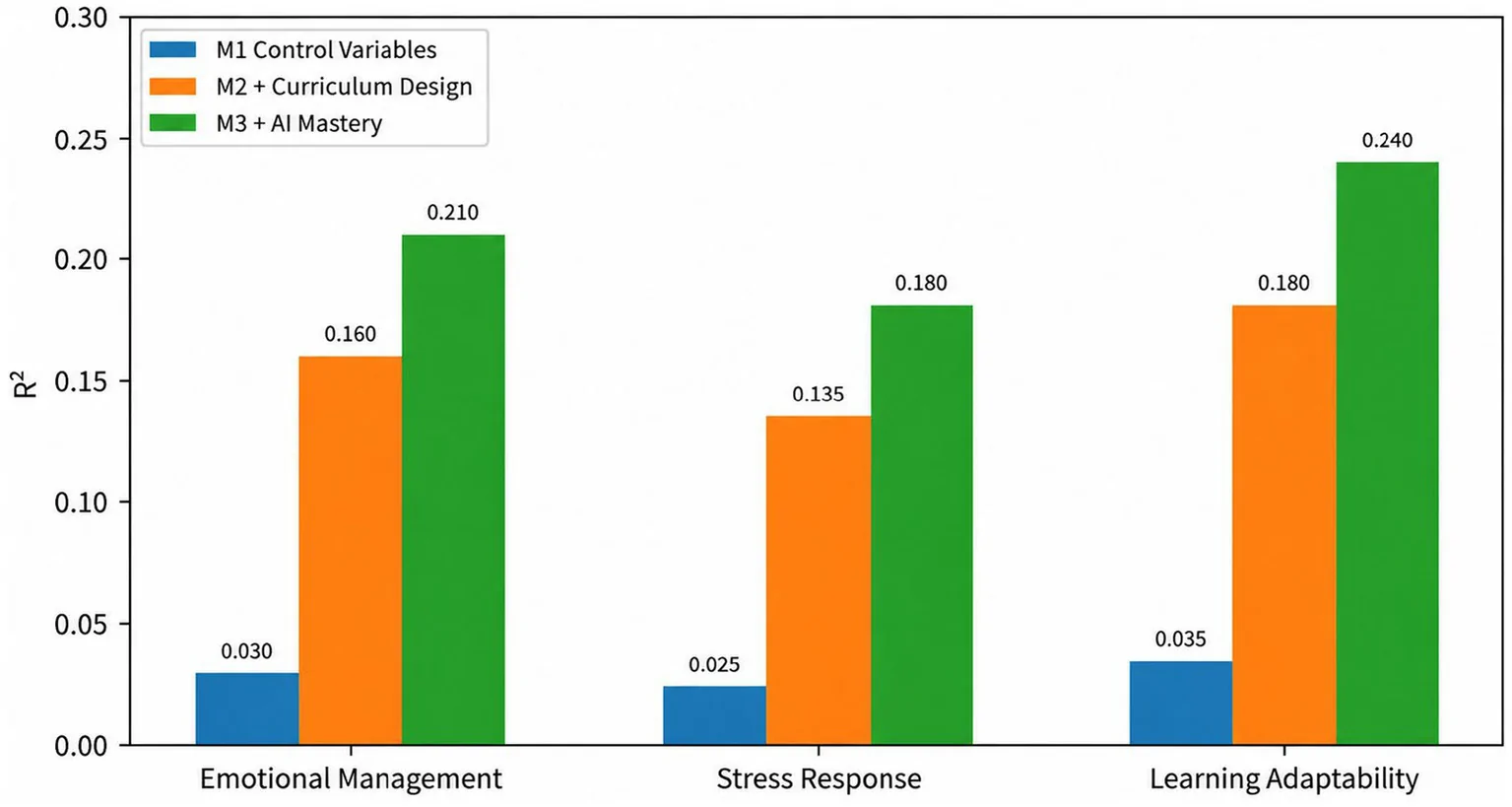
Gradual improvement in the explanatory power of the hierarchical regression model.

Table 5 presents the hierarchical regression analyses. In terms of incremental variance, course development satisfaction had its largest Δ*R*^2^ for academic adjustment (Δ*R*^2^ = 0.145); AI proficiency also had its largest Δ*R*^2^ for academic adjustment (Δ*R*^2^ = 0.060). This further indicates that the most direct statistical association of AI-enabled course development with self-reported psychological adjustment in this sample is on students’ adaptation to university learning methods and the AI learning environment.

**Table 5:** Summary of the hierarchical regression model.

Dependent variable	Model	Enter variable	*R* ^2^	Δ*R*^2^	Significance
Self-reported emotional management	M1	Demographic covariates (gender, major, housing)	0.030	0.030	*p* < 0.01
Self-reported emotional management	M2	+Course development satisfaction	0.160	0.130	*p* < 0.001
Self-reported emotional management	M3	+Self-reported AI proficiency	0.210	0.050	*p* < 0.001
Self-reported stress coping	M1	Demographic covariates (gender, major, housing)	0.025	0.025	*p* < 0.01
Self-reported stress coping	M2	+Course development satisfaction	0.135	0.110	*p* < 0.001
Self-reported stress coping	M3	+Self-reported AI proficiency	0.180	0.045	*p* < 0.001
Self-reported academic adjustment	M1	Demographic covariates (gender, major, housing)	0.035	0.035	*p* < 0.001
Self-reported academic adjustment	M2	+Course development satisfaction	0.180	0.145	*p* < 0.001
Self-reported academic adjustment	M3	+Self-reported AI proficiency	0.240	0.060	*p* < 0.001

### 6.2 Bootstrap mediation analysis

To test whether AI proficiency mediates the statistical association between course development satisfaction and self-reported psychological adjustment (H3, H4), the Bootstrap method (PROCESS Model 4, 5,000 resamples) was used to test the indirect effect. The results showed that course development satisfaction was positively associated with AI proficiency (path *a* = 0.46, *p* < 0.001); after controlling for course development satisfaction, AI proficiency was positively associated with self-reported psychological adjustment (path *b* = 0.27, *p* < 0.001); and the direct association of course development satisfaction with self-reported psychological adjustment remained significant (*c*′ = 0.29, *p* < 0.001). The indirect effect was 0.124 (Boot SE = 0.026, 95% CI [0.076, 0.181]), which does not include zero, supporting H4: AI proficiency partially mediated the association between course development satisfaction and self-reported psychological adjustment (Figure 8).

**Figure 8:**
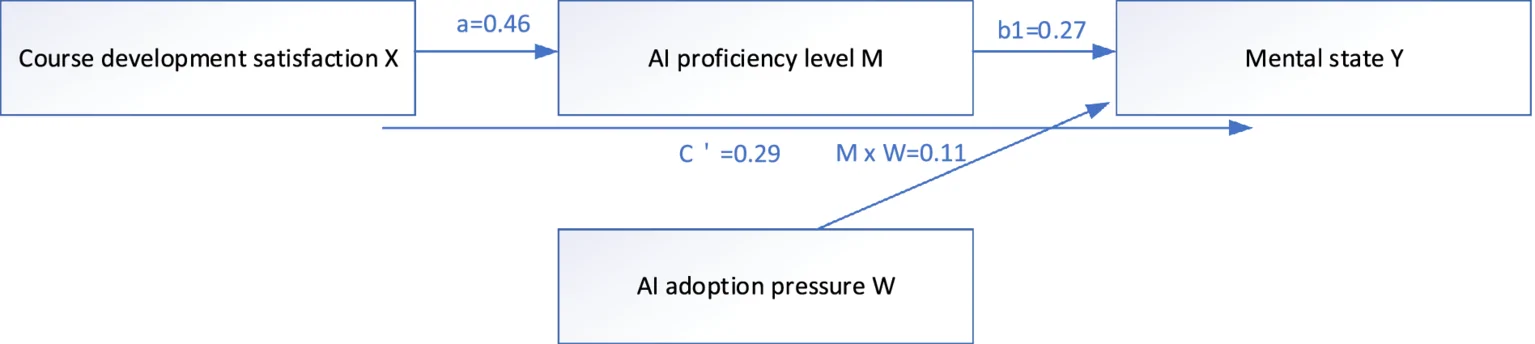
Mediational model of AI proficiency.

Table 6 supports H3 and H4: course development satisfaction not only directly improves students’ self-reported psychological adjustment but also indirectly improves it through enhanced self-reported AI proficiency. It is evident that the positive associations of course development are not solely attributable to direct contextual support but are also transmitted through students’ self-reported AI competencies.

**Table 6:** Bootstrap mediation analysis results.

Path	Effect	Boot SE	Boot LLCI	Boot ULCI	Conclusion
Total effect *c*	0.414	0.043	0.329	0.499	Significant
Direct effect *c*′	0.290	0.040	0.211	0.369	Significant
Indirect effect *a* × *b*	0.124	0.026	0.076	0.181	Significant
Proportion of total effect mediated	30.0%	–	–	–	Partial mediation

### 6.3 The boundary effect of AI proficiency

To test H5, namely whether AI proficiency moderates the association between course development satisfaction and academic adjustment, a simple moderation model (PROCESS Model 1) was estimated. The regression model included course development satisfaction, AI proficiency, and their interaction term. The interaction term between course development satisfaction and AI proficiency was positive and statistically significant (*B* = 0.12, SE = 0.04, *t* = 3.01, *p* = 0.003), indicating that the positive association between course development satisfaction and academic adjustment was significantly stronger among students with higher AI proficiency (Figure 9; Table 7).

**Figure 9:**
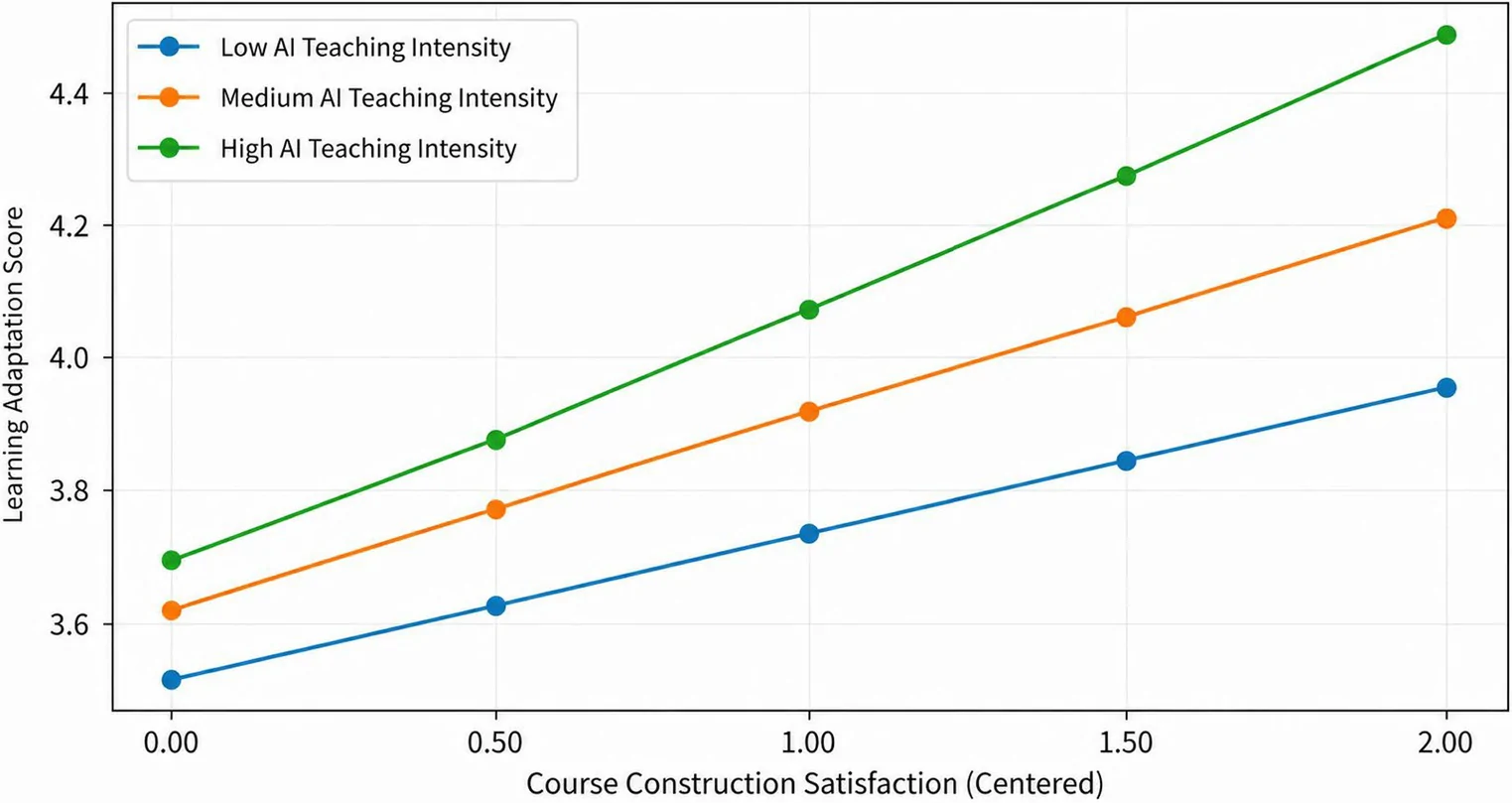
The moderating effect of AI proficiency on the impact of course design on learning adaptation.

**Table 7:** Regression results for moderation effects.

Predictor variables	*B*	SE	*t*	*p*	Interpretation
Course development satisfaction	0.34	0.04	8.10	<0.001	The main effect is significant
Self-reported AI proficiency	0.27	0.04	6.78	<0.001	The main effect is significant
Course development × AI proficiency interaction	0.12	0.04	3.01	0.003	The moderation effect is significant

### 6.4 Moderating and mediating effects of AI usage pressure

To test H6, namely whether AI usage pressure attenuates the positive association between AI proficiency and self-reported psychological adjustment, a moderated mediation model was estimated using PROCESS Model 14. In this model, course development satisfaction was specified as the antecedent of AI proficiency, AI proficiency as the mediator of self-reported psychological adjustment, and the path from AI proficiency to self-reported psychological adjustment as moderated by AI usage pressure.

The results showed that self-reported AI proficiency was positively associated with self-reported psychological adjustment (*B* = 0.31, *p* < 0.001), AI usage pressure was negatively associated with self-reported psychological adjustment (*B* = −0.28, *p* < 0.001), and the interaction term between AI proficiency and AI usage pressure was negative and statistically significant (*B* = −0.11, *p* = 0.004). The simple-slope analysis indicated that the positive association between AI proficiency and self-reported psychological adjustment was significant under low AI usage pressure (1 SD below the mean; simple slope *B* = 0.42, *p* < 0.001), remained significant but weaker under moderate AI usage pressure (simple slope *B* = 0.31, *p* < 0.001), and became non-significant under high AI usage pressure (1 SD above the mean; simple slope *B* = 0.20, *p* = 0.052). Conditional indirect effects indicated that under low AI usage pressure, the indirect association of course development satisfaction with self-reported psychological adjustment through AI proficiency was 0.161 (95% CI [0.099, 0.238]); under moderate pressure, 0.124 (95% CI [0.076, 0.181]); and under high pressure, 0.088 (95% CI [0.035, 0.151]). The moderated mediation index was −0.051 (95% CI [−0.095, −0.017]), which does not include zero, supporting H6: the indirect association between course development satisfaction and self-reported psychological adjustment through AI proficiency was significantly attenuated by AI usage pressure (Figure 10).

**Figure 10:**
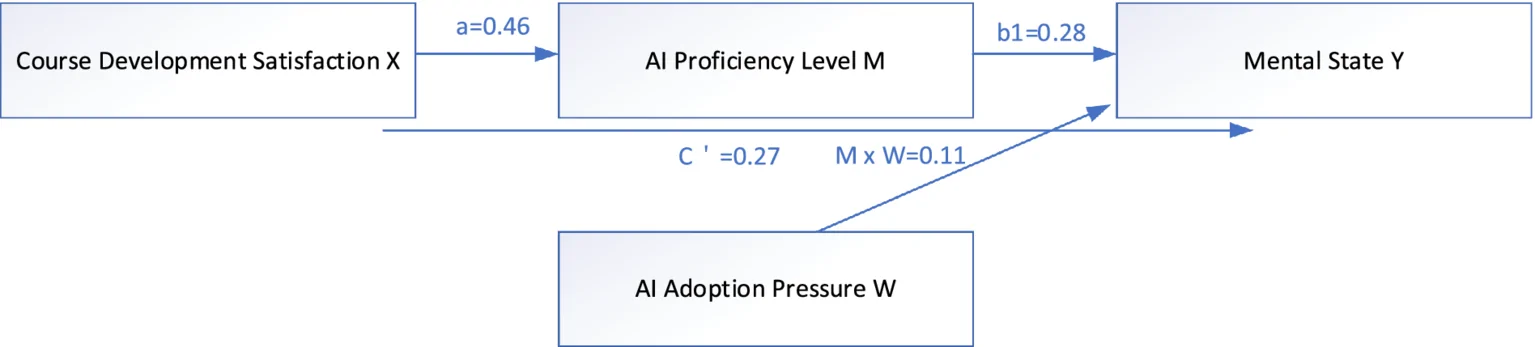
AI-based pressure regulation intermediate pathway.

Table 8 shows that the higher the self-reported AI usage pressure.

**Table 8:** Indirect effects of conditions in PROCESS Model 14.

Self-reported AI usage pressure	Indirect effect	Boot SE	Boot LLCI	Boot ULCI	Interpretation
Low (M − 1 SD)	0.161	0.035	0.099	0.238	The strongest indirect effect
Moderate (M)	0.124	0.026	0.076	0.181	The indirect effect is significant
High (M + 1 SD)	0.088	0.029	0.035	0.151	The indirect effect is attenuated
Moderated mediation index	−0.051	0.020	−0.095	−0.017	Confirmed

### 6.5 AMOS structural equation model

To verify the fit of the overall theoretical model and to complement the regression-based mediation and moderation tests with a latent-variable specification, this study further constructed an AMOS structural equation model. In the model, course development satisfaction, AI proficiency, and self-reported psychological adjustment were all treated as latent variables. Course development satisfaction was measured by observed indicators of course clarity, resource support, and instructor guidance; AI proficiency by observed indicators of AI understanding, AI efficiency, and AI norms; and self-reported psychological adjustment by observed indicators of emotion regulation, stress coping, and academic adjustment (Figure 11; Tables 9, 10).

**Figure 11:**
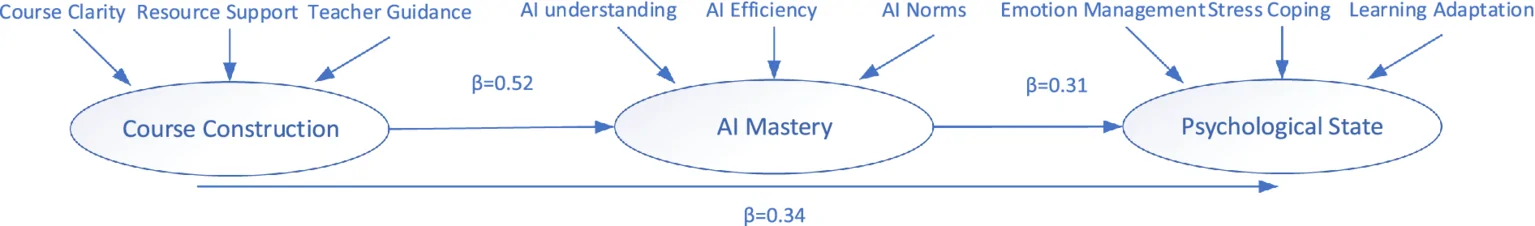
Schematic diagram of the AMOS structural equation model paths.

**Table 9:** AMOS model fit statistics.

Fit index	Results	Recommended threshold	Evaluation
*χ*^2^/df	2.31	<3.00	Acceptable
CFI	0.956	>0.90	Acceptable
TLI	0.941	>0.90	Acceptable
RMSEA	0.049	<0.08	Acceptable
SRMR	0.041	<0.08	Acceptable

**Table 10:** AMOS standardized path coefficients.

Path	Standardized *β*	SE	CR	*p*	Interpretation
Course development → Self-reported AI proficiency	0.52	0.05	10.40	<0.001	Course development significantly positively associated with AI proficiency
AI proficiency → Self-reported psychological adjustment	0.31	0.06	5.17	<0.001	AI proficiency significantly positively associated with self-reported psychological adjustment
Course development → Self-reported psychological adjustment	0.34	0.05	6.80	<0.001	Course development remains directly associated with self-reported psychological adjustment

## 7 Results analysis and discussion

### 7.1 Explanation of the coexistence of “high course approval” and “high psychological stress”

The data show that, while students reported a high level of approval for AI-enabled course development and AI learning tools, their self-reported psychological strain remained equally high. This coexistence indicates that course development and technical support are not, by themselves, sufficient to eliminate the adaptation pressures reported by freshmen. First-year students face a complex adaptation challenge: they must simultaneously understand university coursework, establish professional identity, and navigate interpersonal relationships, while also learning AI tools, adhering to AI guidelines, and completing platform-based assignments. Consequently, even within generally well-designed courses, students may continue to report anxiety and strain because of the accumulation of multiple tasks.

This finding offers insights into the optimistic tendencies of students and faculty in AI-dominated learning environments. While AI tools can indeed enhance learning efficiency, when their use is tied to assignment grading, academic standards, platform tasks, and faculty requirements, students report not only increased efficiency but also additional responsibilities and uncertainty. The key to course design lies not in simply adding AI tools, but in reducing students’ sense of uncertainty through structured design.

### 7.2 Course design as foundational environmental support

Both the regression and structural equation models indicate that satisfaction with course design has a stable, direct positive statistical association with self-reported psychological adjustment. Clear course objectives, adequate resource support, timely faculty guidance, and explicit AI guidelines can help students form stable expectations, enhancing their sense of learning control and psychological safety. For first-year students, the value of course design lies not only in knowledge transfer but also in helping students understand “how to learn in college” and “how to use AI appropriately.”

The statistical association of course design satisfaction generally outweighs that of AI proficiency in the present sample, indicating that contextual support remains the foundation associated with freshmen’s self-reported psychological adjustment. Even if students possess a certain level of AI proficiency, they may still report anxiety and strain if course requirements are confusing, resources are scattered, faculty feedback is insufficient, or guidelines are unclear. Therefore, when universities advance AI course development, they should avoid shifting responsibility entirely to students’ individual abilities and instead strengthen institutionalized support at the course level.

### 7.3 AI proficiency as a competency-based psychological resource

Self-reported AI proficiency plays a partial mediating role between course design satisfaction and self-reported psychological adjustment, indicating that AI competence is one mechanism through which students may transform external course support into internal psychological resources. The more students report understanding AI tools, using AI appropriately, verifying AI outputs, and integrating AI into their learning plans, the more likely they are to report a sense of competence and self-efficacy, which in turn is associated with better emotional management, stress coping, and academic adjustment.

The mediating role of self-reported AI mastery also indicates that the effectiveness of curriculum development can only be fully realized through the development of student competencies. Teachers may be able to enhance students’ self-reported AI mastery-and consequently their self-reported psychological adjustment-by providing AI demonstrations, clarifying task requirements, explaining AI guidelines, and designing progressive AI learning activities. In other words, AI literacy education should be an integral component of first-year curriculum development, rather than merely an add-on tool training.

### 7.4 Risk mechanisms of AI usage stress

PROCESS Model 14 shows that self-reported AI usage pressure attenuates the positive statistical association of AI proficiency with self-reported psychological adjustment. This indicates that even if students report high AI proficiency, this proficiency may not fully translate into self-reported psychological benefits if they face excessive AI tasks, complex platform operations, unclear evaluation criteria, or anxiety stemming from AI usage norms. The association between technical proficiency and self-reported psychological adjustment is not linear but is constrained by the technological stress environment.

This finding offers direct insights for higher education curriculum reform: AI course development should not focus solely on tool coverage and platform usage frequency, but also on students’ technological workload and self-reported psychological adjustment. Excessive reliance on platforms, an overabundance of tasks, and excessive monitoring may transform AI from a learning support into a source of strain. Instructors should control the number of AI-based tasks in course design, clearly define which tasks are suitable for AI use and which must be completed independently, and provide actionable guidelines.

### 7.5 Special implications for first-year students’ academic adjustment

The learning adjustment model demonstrated the highest explained variance, indicating that the most direct statistical association of course design and AI proficiency with self-reported psychological adjustment in this sample lies in the transition of learning styles. As first-year students are new to university, they are not yet fully familiar with academic disciplines, classroom expectations, assignment formats, and evaluation criteria. AI tools can either help them quickly adapt to the learning environment or leave them feeling overwhelmed by information overload and tool dependency.

Therefore, AI course design for first-year students should be transitional and supportive. Courses should not assume that students already possess mature AI learning skills; instead, they should provide step-by-step guidance in areas such as foundational AI literacy, prompt design, source verification, academic integrity, study planning, and stress management. Only in this way can AI tools be reliably associated with first-year students’ academic adjustment in cross-sectional self-report data.

## 8 Conclusion

Based on 551 cross-sectional self-report questionnaires from first-year students, this study examined the statistical associations among AI-enabled course development, self-reported AI proficiency, self-reported AI usage pressure, and self-reported psychological adjustment. The descriptive findings indicate that, while students generally rated course development and AI learning tools highly, self-reported indicators of psychological strain and AI-related task pressure were equally prominent, revealing a coexistence of “high support and high strain” in this sample.

Course development satisfaction and self-reported AI proficiency were both statistically and positively associated with emotional management, stress coping, and academic adjustment. Self-reported AI proficiency partially mediated the association between course development satisfaction and self-reported psychological adjustment, suggesting that course design may translate into psychological benefits through students’ self-reported technical capabilities. Self-reported AI proficiency also moderated the positive association between course development satisfaction and academic adjustment, indicating that self-reported technical competence is one boundary condition for students’ ability to translate course resources into academic adjustment. Conversely, self-reported AI usage pressure attenuated the positive association between AI proficiency and self-reported psychological adjustment, highlighting that technical stress is a risk factor that cannot be overlooked in AI course design.

Overall, the development of AI-enabled courses should follow the “course support-AI literacy-psychological support” trinity principle. Universities should not merely pursue AI tool coverage but should also attend to course structure clarity, the quality of faculty guidance, AI ethics education, workload management, and students’ self-reported psychological adjustment. Because the present design is cross-sectional and self-report-based, the current findings should be interpreted as statistical associations rather than causal effects; longitudinal, multi-institution, and mixed-methods studies are needed before any firm policy recommendations can be drawn.

### 8.1 Limitations and future research

The sample was drawn from a single institution and was predominantly male and engineering-major, which limits generalizability; female students and students from non-engineering majors were under-represented, and the single-institution convenience sample does not capture variation across institutional cultures, regional policy environments, or disciplines. Future studies should adopt multi-institution, stratified, and probability-sampling designs with balanced gender and discipline distributions. The conceptual overlap among psychological state, mental health, academic adjustment, emotional regulation, and stress coping was addressed by treating them as a single self-reported adjustment composite, an operational choice that preserves statistical power but obscures construct-specific effects; future research should examine each sub-dimension separately and validate them against established instruments such as the GAD-7, the PHQ-9, or domain-specific academic-adjustment scales.

## Data Availability

The raw data supporting the conclusions of this article will be made available by the authors, without undue reservation.
